# Broad consent under the GDPR: an optimistic perspective on a bright future

**DOI:** 10.1186/s40504-019-0096-3

**Published:** 2020-01-06

**Authors:** Dara Hallinan

**Affiliations:** FIZ Karlsruhe – Leibniz-Institut für Informationsinfrastruktur, Hermann-von-Helmholtz-Platz 1, 76344 Eggenstein-Leopoldshafen, Germany

**Keywords:** Genomics, Genetics, Genomic research, Biobank, Medical research, Consent, Broad consent, Data protection law, Privacy law, General data protection regulation

## Abstract

Broad consent – the act of gaining one consent for multiple potential future research projects – sits at the core of much current genomic research practice. Since the 25th May 2018, the General Data Protection Regulation (GDPR) has applied as valid law concerning genomic research in the EU and now occupies a dominant position in the legal landscape. Yet, the position of the GDPR concerning broad consent has recently been cause for concern in the genomic research community. Whilst the text of the GDPR apparently supports the practice, recent jurisprudence contains language which is decidedly less positive. This article takes an in-depth look at the situation concerning broad consent under the GDPR and – despite the understandable concern flowing from recent jurisprudence – offers a positive outlook. This positive outlook is argued from three perspectives, each of which is significant in defining the current, and ongoing, legitimacy and utility of broad consent under the GDPR: the principled, the legal technical, and the practical.

## Introduction^1^

Broad consent is a form of consent used in genomic research which permits genomic researchers to collect biological samples, genomic data and other research subject data for use in unspecified future research projects. The benefit of such a process is clear. It allows genomic research to proceed on the basis of research subject consent whilst providing optimum utility for research – research materials can unproblematically be used in new projects, even with novel research protocols, whilst greater total collections of samples and data can be mobilised to facilitate larger and more accurate research. It is thus no surprise that broad consent is employed by a large, and growing, number of genomic research infrastructures in Europe.

The General Data Protection Regulation (GDPR) has applied since 25th May 2018 (European Parliament and Council 2016). The GDPR is EU level legislation directly applicable in all EU Member States providing citizens with protection whenever their personal data are processed. Its provisions apply almost completely across the genomic research process – from the moment of collection of biological samples and associated data up until the production of research results. It is not an exaggeration to state that the GDPR now occupies a dominant position in the hierarchy of legal instruments governing genomic research in the EU. It is also not an exaggeration to state that the arrival of the GDPR has been accompanied by uncertainty and unease in the genomic research community.

Four years ago, during the legislative process leading up to the GDPR, an article was published – in this journal – considering the legitimacy of broad consent in genomic research under the GDPR (Hallinan and Friedewald 2015). This article suggested the outlook for the legitimacy of broad consent under the GDPR, at that time, was poor. As the legislative process moved on, however, support for broad consent in the EU institutions appeared to grow and, as a result, significant changes were made to initially problematic provisions. Consequently, the final text of the GDPR now contains provisions which seem tailor made to support broad consent. Reading the final text of the GDPR, all seems well.

However, in the period between the adoption of the final text of the GDPR and now, the Article 29 Working Party – the body tasked with providing EU level guidance, interpretation and adaptation of the provisions of GDPR – chose to weigh in on the legitimate scope of consent in the GDPR in relation to scientific research. The content of their guidance is, superficially at least, not friendly to the practise of broad consent. The guidance has thus been, understandably, cause for concern in the genomic research community. Whilst recognising the legitimacy of these concerns, this article will offer a more optimistic perspective and will highlight the reasons for a positive outlook on the current and ongoing legitimacy and utility of broad consent under the GDPR.^2^

The article begins by offering an overview of broad consent – process, justification and uptake (section 1). Next, the article will elaborate why the position of the GDPR on broad consent matters (section 2). Consequently, the article will offer an overview of the law and jurisprudence concerning broad consent under the GDPR – including Article 29 Working Party guidance – and will specify why recent jurisprudence has caused concerns (section 3). Whilst recognising the legitimacy of these concerns, the article finally outlines arguments for a positive outlook. Arguments are clustered into three perspectives: the principled legitimacy of broad consent (section 4); the legal technical legitimacy of broad consent (section 5); and the practise of broad consent irrespective of data protection law under the GDPR (section 6).

### Broad consent: a brief overview

To begin, a brief overview of the practise of broad consent is necessary. Such an overview can usefully be provided from three perspectives: the process and scope of broad consent; the utility and justification of broad consent; and the support and uptake of broad consent.

In order to conduct genomic research using competent adults’ biological samples and associated data – genomic, health and lifestyle data – researchers may be obliged to seek consent. Broad consent is one type of consent researchers may seek (Hallinan and Friedewald 2015, pp. 4–6). In broad consent, the research subject need only engage with the genomic researcher – or genomic research infrastructure – once. In this engagement, the research subject may be asked for permission concerning – as necessary depending on context – three types of activity:

1. The extraction of their biological sample and the collection of relevant associated data – including genomic, health and lifestyle data.
2. The storage – and as required subsequent preparation – of their biological sample and any associated data for use in research.
3. The future use of their collected biological sample and associated data for – at least at the time of collection – unspecified research purposes (Hallinan and Friedewald 2015, p. 5).

Broad consent is specific to genomic research. Broad consent differs significantly from previous concepts of consent used in clinical medical research which tended to require the research subject to provide consent only to specifically elaborated research projects (Biggs 2009, pp. 17–35).

The rationale behind broad consent is clear. On the one hand, broad consent allows genomic researchers to engage in research based on consent. In doing so, broad consent allows genomic researchers to engage in research for which research subjects have expressed an autonomous wish to participate. In some cases, obtaining consent may be the only legally permissible way to engage in such research. In all cases, obtaining consent represents an optimum confluence between potentially conflicting interests in the research process – particularly those of the research subject and the genomic researcher. On the other hand, broad consent offers maximum utility to the genomic research mission. This is true for three reasons in particular. First, broad consent allows each collected sample and associated data set to be used for multiple research purposes without the obligation to recontact research subjects to request new permissions for each new project – with the administrative and resource allocation this would require (Sheehan 2011, p. 226). Second, broad consent permits each sample and associated data-set to be used in truly novel research projects, with novel research protocols which could not have been – owing to the speed of progress of genomic research methodologies – foreseen at the moment of collection. Finally, broad consent permits the assembly of greater total quantities of samples and data available for research and thereby facilitates genomic research projects of greater scale.

Broad consent already has considerable backing in law and practise in Europe. In terms of law, a consideration of the position of normatively significant international instruments with relevance to European genomic research is enlightening: all such international instruments drafted over the past decade highlight the legitimacy of broad consent. See, for example: the World Medical Association’s Declaration of Taipei – Article 12 (World Medical Association 2016); the Organisation for Economic Cooperation and Development’s Guidelines on Human Biobanks and Genetic Research Databases – Article 4.6 (Organization for Economic Co-Operation and Development 2009); and the Council of Europe’s Recommendation CM/Rec (2016)6 of the Committee of Ministers to member States on research on biological materials of human origin – Article 11 (Council of Europe 2016). The legitimacy of broad consent has also been explicitly recognised in EU Member State law. See, for example: the Estonian Human Genes Research Act – Article 12 (Riigikogu 2000); and the UK’s Human Tissue Act – as elaborated by the Human Tissue Authority’s guidelines on consent (UK Parliament 2004; Human Tissue Authority 2017, p. 11). In terms of practise, the lack of empirical research on consent practises in genomic research makes clear identification of trends in the use of broad consent difficult to identify (Hallinan 2018, p. 77). Nevertheless, it is indicative that experts, such as Strech et al., observe that: ‘[genomic research] increasingly presume[s] long-term storage of biomaterials and data that shall be used for future research projects which are today unspecified’ (Strech et al. 2016, p. 295). In such constellations, broad consent is the usual approach to legitimating research activity.

Given the clear benefits to broad consent, it is unsurprising the practise has had considerable, and indeed increasing, uptake in law and practise. The legal situation concerning broad consent in genomic research in the EU, however, is constantly evolving. Recently a new, and highly significant, EU law has become applicable to genomic research: the GDPR. The GDPR has been in force since 25th of May 2016 and has applied as valid law for genomic research since 25th of May 2018 – as foreseen in Article 99 (2). The GDPR is an EU Regulation, which means its provisions are directly binding – unless explicitly specified – in all EU Member States and supersede existing Member State law. The GDPR arguably applies across the genomic research process whenever personal data are processed – including to the collection, storage and use of research subjects’ biological samples, genomic data and other associated data (Hallinan 2018, pp. 263–295; Hallinan and De Hert 2016).^3^ Prior to considering the legal details of the GDPR’s stance on broad consent, however, it is worth considering the importance of this stance for the legitimacy and utility of broad consent. For this, it is necessary to consider the GDPR’s approach to the legitimation of genomic research generally.

### Legitimating genomic research under the GDPR: the importance of the position of the GDPR on broad consent

One of the key tenets of the GDPR, outlined in Articles 9 (1) and (2), is that all processing of sensitive personal data – a category covering all personal data processed in genomic research – requires justification (Hallinan 2018, pp. 305–308).^4^ The range of legitimate justifications are exhaustively listed in Article 9 (2).^5^ Accordingly, in order to be legitimate and to comply with the GDPR, genomic research must be able to point to one of these justifications as relevant. Only three justifications – out of ten – reasonably apply to genomic research: Article 9 (2)(a), Article 9 (2)(g) and Article 9 (2)(j). The specifics of these justifications, and more importantly the relationship between them, should be considered in more detail.

To start, it is worth sketching the scope, and the general conditions of use, of each of the three justifications relevant for legitimating the processing of sensitive personal data in genomic research:

1. Article 9 (2)(a): the Article legitimates the processing of sensitive personal data in genomic research if: ‘the data subject has given explicit consent to the processing of those personal data for one or more specified purposes’.^6^ A consent will only be legitimate, however, if it fulfils subsequent conditions outlined in Article 4 (11). These conditions require consent to be: freely given – the research subject must not be forced; specific – the consent should only relate to a certain scope of processing; informed – adequate information permitting understanding of the facts and consequences of processing must be communicated to the research subject in the consent process; and unambiguous – the desire to consent should be unmistakably indicated.^7^
2. Article 9 (2)(g): the Article legitimates the processing of sensitive personal data in genomic research for: ‘reasons of substantial public interest’. The justification may only be relied upon, however, if two subsequent criteria are fulfilled. First, Article 9 (2)(g) clarifies that the genomic research must be elaborated in a ‘Union or Member State law which … provide [s] for suitable and specific measures to safeguard the fundamental rights and the interests of the data subject’. Second, relevant jurisprudence suggests that the genomic research must be explicitly elaborated as a ‘substantial’ public interest in Member State law (Article 29 Working Party 2005, pp. 14–15).^8^
3. Article 9 (2)(j): the Article permits the processing of sensitive personal data for genomic research provided this is necessary for: ‘archiving purposes in the public interest, scientific or historical research purposes’. This justification may only be relied upon, however, if two subsequent criteria, also outlined in Article 9 (2)(j), are fulfilled. First, certain measures, explicitly outlined in Article 89 (1), aimed at ensuring research subject rights, must be in place – these measures include pseudonymisation and adequate data protection by design and default measures. Second, the genomic research must be elaborated in a ‘Union or Member State law … which provides for suitable and specific measures to safeguard the rights and freedoms of the data subject’.^9^

In terms of relationship – whilst not explicitly elaborated in the GDPR – the three justifications can be argued to exist in a two-level hierarchy, with consent under Article 9 (2)(a) at the top. Accordingly, whenever consent, under Article 9 (2)(a), can be relied on as a legitimation for the processing of sensitive personal data in genomic research, this should be preferred to reliance on a legitimation under Article 9 (2)(g) or (j). The existence of such a hierarchy can be argued in terms of law. The existence of such a hierarchy can also be argued in terms of practical utility.

Two principled legal arguments for the existence of a hierarchy can be put forward.^10^ First: the GDPR constitutes a process through which research subjects’ fundamental rights are effectively balanced against interests tied up with the genomic research process. From a fundamental rights perspective, where sensitive data are processed on the basis of consent – under 9 (2)(a) – there is no, prima facie, infringement of the research subject’s fundamental rights. When sensitive personal data are processed under all other justifications – including Articles 9 (2)(g) and 9 (2)(j) – an infringement is, prima facie, present, albeit legitimated. According to fundamental rights law, the justification constituting the lesser rights infringement – consent – must, all things being equal, be preferred (Beyleveld 2004, p. 12). Second, the GDPR is omnibus legislation requiring substantive clarification in relation to specific processing sectors – such as in relation to which Article 9 (2) justification should be sought in genomic research (Hallinan 2018, p. 386). This substantive clarification should be informed by – ethical and legal – norms present in the context in question. A dominant norm that consent should be sought whenever possible is already identifiable in relation to genomic research. This is the stance, for example, of all international genomic research instruments with European relevance – see for example, those discussed above, in section 1. It is true these principled justifications remain academic arguments – rather than settled jurisprudence. Nevertheless, their legal logic is persuasive and there is little work identifiable which either highlights their flaws or provides substantial counter-argumentation.

Two practical utility arguments can also be put forward. First, whilst 9 (2)(a) is always usable, it is not the case that Articles 9 (2)(g) and (j) will always be usable. In relation to Article 9 (2)(g), it is not necessarily the case that all Member States’ laws will recognise genomic research as serving a ‘substantial’ public interest – and even if they do, it is not certain that these laws will extend the recognition to all forms of genomic research. In such Member States, Article 9 (2)(g) may not confidently be relied on.^11^ In relation to both Articles 9 (2)(g) and (j), not all Member States currently have legislation outlining ‘suitable and specific’ safeguards in relation to genomic research. Germany, for example, arguably has no such genomic research ‘specific’ legislation (Hoppe 2016, 35–44). In such Member States, neither justification may confidently be relied on. Second, even in Member States in which Articles 9 (2)(g) and (j) can, in principle, be relied on to legitimate genomic research, the more these justifications are relied on in practise, the more genomic research will differ between Member States – by virtue of the need to follow nationally specific legal obligations. This risks a fragmentation of approaches to genomic research and the awkward erection of legal and administrative barriers to cross-border research. This would act contrary to one of the key opportunities of the GDPR for genomic research: harmonisation of EU research standards (Research and Patient Organisations 2016, p. 1).

The discussion in the section above clarified that all processing of sensitive personal data in genomic research requires a justification under the GDPR. The discussion also clarified that consent, under Article 9 (2)(a), is preeminent – both legally and practically – amongst all applicable justifications. Thus, the degree to which the conditions of a legitimate consent under 9 (2)(a) permit broad consent will be definitive of the general legitimacy and utility of broad consent. We thus come to the key question: is broad consent legitimate under Article 9 (2)(a)? There has recently been some, understandable, concern that this might not be the case.

### Concern as to the legitimacy and utility of broad consent under article 9 (2)(a) GDPR

In principle, broad consent can fulfil, unproblematically, all but one of the conditions for a legitimate consent under Article 9 (2)(a) – in conjunction with Article 4 (11). The potentially problematic condition relates to the need for specificity in the scope of consent. In prior data protection jurisprudence, the general concept of specific consent had been seen as being in direct conflict with broad consent (Article 29 Working Party 2013, p. 16; Hallinan and Friedewald 2015, pp. 10–16). Fortunately for broad consent, the text of the GDPR includes further clarifications which endorse the relaxation of conditions of specificity in relation to scientific research and which apparently support broad consent. Unfortunately for broad consent, however, recent jurisprudence can be interpreted as signalling a move back toward the need for specific consent in scientific research. Naturally, this jurisprudence has been cause for concern in the genomic research community.

The final text of the GDPR – in part as a result of lobbying by genomic research organisations – includes provisions which seem to offer clear support for broad consent (Thompson 2016). Specifically, Recital 33 relaxes the general Article 4 (11) specificity requirements in relation to the permissible scope of consent in scientific research – including in relation to Article 9 (2)(a). The Recital states: ‘It is often not possible to fully identify the purpose of personal data processing for scientific research purposes at the time of data collection. Therefore, data subjects should be allowed to give their consent to certain areas of scientific research when in keeping with recognised ethical standards for scientific research. Data subjects should have the opportunity to give their consent only to certain areas of research or parts of research projects to the extent allowed by the intended purpose.’ It is true there are uncertainties in the text of Recital 33.^12^ Nevertheless, the presence of the Recital makes the assertion that the GDPR supports broad consent the logically defensible position. This is a defensible position from a legal technical perspective. A straightforward textual reading of the Recital clearly indicates support for the legitimacy of broad consent. This is also a defensible position from a normative perspective. An interpretation of Recital 33 supporting broad consent matches the position of the GDPR to the dominant normative approach in biomedical research law – see, for example, the norm for broad consent across all international instruments with European relevance dealing with genomic research, discussed in section 1. Consequently, this interpretation of Recital 33 has been generally recognised as correct by those commenting on genomic research and the GDPR. Rumbold and Pierscionek, for example, confidently assert: ‘the agreed text permits broad consent’ (Rumbold and Pierscionek 2017, p. 2).

Following the adoption of the GDPR, however, in 2017 supplemental guidance concerning the specificity of the consent requirement in relation to scientific research was provided by the Article 29 Working Party. Unfortunately, the Working Party’s ‘Guidelines on consent under Regulation 2016/679’ contain much less favourable pronouncements on Recital 33 and broad consent. Two aspects of the Working Party’s guidance can be read as particularly problematic for broad consent. First, the guidance seems to aim to restrict the scope of applicability of Recital 33 and thus of broad consent. The guidance states ‘First, it should be noted that Recital 33 does not disapply the obligations with regard to the requirement of specific consent. This means that, in principle, scientific research projects can only include personal data on the basis of consent if they have a well-described purpose. For the cases where purposes for data processing within a scientific research project cannot be specified at the outset, Recital 33 allows as an exception that the purpose may be described at a more general level.’ (Article 29 Working Party, 2017, p. 28). Second – in relation to those cases to which Recital 33 would still remain applicable – the guidance seems to endorse the need for subsequent rolling granular consents over one, *ex ante,* broad consent: ‘When research purposes cannot be fully specified, a controller must seek other ways to ensure the essence of the consent requirements are served best, for example, to allow data subjects to consent for a research purpose in more general terms and for specific stages of a research project that are already known to take place at the outset. As the research advances, consent for subsequent steps in the project can be obtained before that next stage begins’ (Article 29 Working Party, 2017, p. 28).^13^ These pronouncements have caused, quite understandably, concern in the genomic research community as to the ongoing legitimacy and utility of broad consent under the GDPR (BBMRI-ERIC 2017; Kubetin et al. 2018).

Those unfamiliar with the institutions of data protection law may wonder why this guidance is so significant. The answer comes in a consideration of the pedigree of the issuing body. The Article 29 Working Party was the body tasked with the EU level interpretation of data protection law under the forerunner to the GDPR – Directive 95/46. The group consisted of representatives of the national Data Protection Authorities (DPAs) – the institutions charged with interpreting and enforcing data protection law in EU Member States. With the applicability of the GDPR in 2018, the Working Party was officially succeeded by the European Data Protection Board (EDPB). Whilst the EDPB officially succeeds the Working Party, however, much remains substantially unchanged. The EDPB has the same role as the Article 29 Working Party, is populated by the same key Member State actors as the Working Party and has adopted all the Working Party’s recent opinions and guidance documents – including the problematic guidance on consent (European Data Protection Board 2018, p. 1). It is true that Article 29 Working Party and EDPB guidance is, by the letter of the law in Article 70 GDPR, non-binding. However, such interpretative guidance – as a result of both the composition and legal position of the bodies – has come to occupy a vaunted position in data protection jurisprudence. Indeed, the importance of such guidance is, arguably, only superseded by the text of the GDPR and case-law. Further, the EDPB does have the power to translate guidance, as necessary, into binding proclamations – for example under Article 65 GDPR. As De Hert and Papakonstantinou argue, this is a: ‘strong and standalone Board … capable of deciding … and enforcing … opinions’ (De Hert and Papakonstantinou 2016, p. 193).

Given the content of the guidance and the status of the issuing body, it is unsurprising that concern has been voiced in the genomic research community. Despite the fact that such concern is warranted, however, there are a number of arguments which can be put forward supporting the current and ongoing legitimacy and utility of broad consent under the GDPR. These arguments can be usefully clustered into three perspectives. Whilst it is difficult, *ex ante*, to define the relative significance of each perspective or the mechanics of the interaction of perspectives, it is reasonable to assert that each perspective is significant. The three perspectives are: the principled; the legal technical; and the practical.

### The principled perspective supporting the legitimacy and utility of broad consent under the GDPR (perspective 1)

Each provision in the GDPR seeks to operationalise certain underlying rationale – for example the right to access under Article 15 seeks to ensure the transparency of data processing in relation to the individual whose data is being processed. The phenomena data protection law seeks to regulate – data processing and its social consequences – however, have proven to be conceptually difficult to grasp, to be subject to fast and unpredictable development and to manifest in different ways in different social contexts. These facets of the regulatory phenomena have made it difficult for lawmakers to define monolithic, black letter, data protection provisions capable of sensibly applying, in a harmonised way, across all relevant technologies and contexts. This difficulty is clearly demonstrated in Mayer-Schönberger’s explication of the inadequacies of successive generations of European data protection law to identify monolithic principles capable of keeping pace with technological and social development (Mayer-Schönberger 1997, pp. 219–242). In response to these difficulties, provisions in the GDPR have been designed to be as flexible and adaptable as possible to context, to allow the optimal expression of their underlying rationale despite environmental variation – a reflexive interplay between rationale, provision and context is intended. Accordingly, a key step in clarifying whether a practise might be regarded as legitimate under the GDPR, is to consider whether the rationale behind a practise in a given context aligns with the rationale behind the relevant provisions of the GDPR. Such an alignment can be identified between broad consent in genomic research and consent provisions in the GDPR.

The base rationale behind broad consent in genomic research and behind consent provisions in the GDPR are aligned. As long recognised in the German context, and with increasing recognition at European level – as elaborated by, for example, Lynskey – one of the underlying goals behind data protection law is to provide the individual with informational self-determination: the right to decide when, how and by whom, their personal data are processed (Bundesverfassungsgericht 1983; Lynskey 2015, pp. 177–229). Consent in the GDPR – including under Article 9 (2)(a) – is the concrete mechanism giving voice to this underlying rationale. Logically then, limitations to the ability of the individual to enjoy their right to informational self-determination – and to give their consent to any act of data processing – are in principle undesirable. There is no prima facie reason that limits on consent imposed by the legislator should be treated any differently. This assertion includes legislative limits imposed on the legitimate scope of consent – for example if the Article 29 Working Party’s guidance were to be read as requiring specific consent rather than broad consent in genomic research. It follows from this underlying logic that the concept of consent under the GDPR need not, in principle, directly oppose broader forms of consent – including broad consent in genomic research. Indeed, as Taupitz and Weigel put it, in cases of uncertainty, data protection should support the individual’s right to ‘take a risk’ (Taupitz and Weigel 2012, pp. 265–266).

It is true that there are legitimate second-order reasons which may justify the legislator’s choice to impose limits on the scope of consent in the GDPR (Hallinan 2018, p. 437). Such reasons, however, are not demonstrably relevant in relation to genomic research infrastructures employing broad consent. Three such legitimate reasons for legislator limitations can be highlighted as relevant. First: if there is a significant power imbalance between the individual and the entity processing their data. Second: if the interests of the individual are misaligned with those of the entity processing their data. Third: if a lack of informational clarity in relation to the scope of a consent transaction seems likely to obscure relevant information to the detriment of the individual. Such legitimate reasons are evidently applicable in relation to many commercial and bureaucratic data processing scenarios – for example, online behavioural advertising (Zuiderveen Borgesius 2013, p. 28). It is, however, much harder to see that these legitimate reasons apply to genomic research infrastructures operating broad consent. In relation to the first reason, the dependant relationship necessary to facilitate power imbalance is absent between genomic researchers and research subjects. In relation to the second reason, genomic research is, as Laurie puts it, a collaborative endeavour where the interests of researcher and research subject are ideally aligned (Laurie 2002, p. 167). In relation to the final reason, the lack of information provided concerning the scope of uses possible under broad consent process does not serve to obscure relevant information from the research subject – the information in question is not available to any party. Further, genomic research has no intention to have an impact on single research subjects – the aim is the generation of abstract scientific knowledge (Hallinan 2018, p. 123).

Even if such legitimate reasons were, to some degree, applicable to genomic research, the permissible scope of consent in a given context should still represent a balance between relevant interests. Thus: the final scope of consent in the GDPR as it applies to genomic research should reflect a balance between legitimate reasons to limit the scope of consent to protect the research subject, and the individual and social benefits which might emerge from retaining a broad scope of consent. In the case of genomic research, the most obvious benefits of a broad scope of consent relate to: the generation – via research facilitated by broad consent – of knowledge about gene function and expression and the consequent development, on the back of this knowledge, of better healthcare interventions. The prospective benefits of genomic research have been asserted for many years as a justification for broad consent – for example, by Hansson et al. over a decade ago (Hansson et al. 2006, p. 267). Empirically, however, such assertions were not always validated by concrete data. Writing in 2012, for example, Visscher et al. provided reason to doubt the delivery of such benefits via genomic research – both in terms of the quantity of useful knowledge generated as well as in terms of the totality of health interventions developed (Visscher et al. 2012, pp. 7–24). More recently, however, such doubts have been shown to be unwarranted. The benefit argument is now supported by a solid empirical base. Visscher et al., writing in 2017, revisited their prior conclusions and now present clear evidence for the benefits of genomic research (Visscher et al. 2017, pp. 5–22). They highlight both the enormous quantity of useful knowledge being generated by genomic research as well as the impressive, and ever increasing, quantities of medical interventions developed on the back of this knowledge.

Whilst a principled defence of broad consent is valuable in justifying the legitimacy and utility of the practise under the GDPR, such a defence does not directly engage with the technical details of the law or associated jurisprudence. Accordingly, regardless of the strength of the principled perspective, if this perspective turns out to contradict the technical details of the law, the practise of broad consent would still remain, at the very least, awkwardly disputed.

### The legal technical perspective supporting the legitimacy and utility of broad consent under the GDPR (perspective 2)

Fortunately, in the case of broad consent, an argument can be mounted that no contradiction exists between the practise and the technical details of law. This argument might be mounted from three perspectives: a close reading of the problematic Article 29 Working Party guidelines; a contextual reading of the Article 29 Working Party guidelines; and a consideration of the legal efficacy of any Article 29 Working Party effort to contradict the legitimacy of broad consent.

First, a close reading of the text of the Article 29 Working Party guidelines reveals considerable vagueness. This vagueness offers the potential for the guidelines to be given interpretations which are unproblematic for broad consent. Interpretations ameliorating the consequence of the two key problematic statements – identified in section 3 – might be put forward. First problematic assertion: ‘Recital 33 does not disapply the obligations with regard to the requirement of specific consent’ (Article 29 Working Party 2017, p. 28). This general statement is followed, in the same paragraph, with the exception that: ‘where purposes for data processing within a scientific research project cannot be specified at the outset, Recital 33 allows as an exception that the purpose may be described at a more general level’ (Article 29 Working Party 2017, p. 28). This exception can easily be given a broad interpretation to apply to any relevant, open ended, genomic research process for which broad consent could be beneficial. Second problematic assertion: ‘As the research advances, consent for subsequent steps in the project can be obtained before that next stage begins’ (Article 29 Working Party 2017, p. 29). This general statement is followed, in the next sentence, with the qualification that: ‘such a consent should … be in line with the applicable ethical standards for scientific research’ (Article 29 Working Party 2017, p. 28). There is no indication of an importance hierarchy between these two sentences. Accordingly, there is no reason the latter sentence cannot be interpreted to mean an ethically legitimate consent may be permissible even if this consent would exclude the need for continual reconsent for each step of a project. In this regard, recall – as already highlighted in sections 1 and 4 – that there is already strong support for the ethical and legal legitimacy of broad consent in genomic research.

Second, a contextual reading of the Article 29 Working Party guidelines shows they do not need to be taken as providing an interpretation of the GDPR explicitly relevant for broad consent in genomic research. The GDPR was designed as omnibus legislation, whose substantive provisions were to be adapted, as logical and necessary, to the specifics of each processing context. In this regard, the approach to consent in the Article 29 Working Party’s guidance scarcely differentiates its application to different types of scientific research – it may be regarded as omnibus guidance in relation to scientific research. It can be argued that health research is a special category of scientific research in terms of method, normative legitimacy and result. It can further be argued that genomic research is a special category of health research in terms of method, normative legitimacy and result: no other form of health research aims at the systematic analysis of genome function, expression and genome-environment interaction; no other form of health research promises to provide the stratification of populations needed for the development of precision medicine systems; and no other form of health research supports the development of genetically targeted medical interventions (Hewitt 2011, p. 112). As Karsten et al. observe, genomic research may be seen as a unique construct (Karsten et al. 2011, p. 573). Accordingly, it is legitimate to suggest that the applicability of interpretations of the GDPR addressed to scientific research generally, may be set aside in cases where they create friction with normatively legitimate practises – such as broad consent – unique to genomic research.

Finally, there are boundaries to the powers of the EDPB under the GDPR. In the light of these boundaries, it is questionable whether the Article 29 Working Party’s interpretations of consent, if they are read to undermine the legitimacy and utility of broad consent, can be taken as legally valid. Article 70 of the GDPR outlines the powers of the EDPB. Even in their broadest formulations, when the positions of the EDPB are legally binding – not the case in relation to the non-binding guidelines concerning consent discussed in this article – the scope of these powers is still limited to the interpretation and adaptation of the GDPR (Hallinan 2018, pp. 404–405). The EDPB thus only has the power to act in areas the legislator has left open to interpretation and adaptation. The EDPB does not have the power to move against the express wishes of the legislator. Indeed, for an administrative body such as the EDPB to do so could be seen as undemocratic. Interpretations of the Working Party’s guidance limiting the utility of broad consent could be argued to move against the intentions of the legislator. It is a matter of record that prior versions of the GDPR – those which emerged in the legislative process – proposed the need for specific consent in scientific research. The European Parliament’s version, for example, insisted consent could only extend to ‘one or more specific and similar researches’ (European Parliament 2014, Article 81(1c)). It is also a matter of record that the legislator removed such provisions and replaced them with Recital 33. In this regard, it is most logical to suggest Recital 33, and the final text of the GDPR, support broad consent – as discussed above, in section 3.

Even if both the principled and the legal technical perspectives are discounted as relevant, it is not the case that law alone is necessarily decisive in determining the ongoing utility of a process such as broad consent. The interplay between law and practise is also important.

### The practical perspective supporting the legitimacy and utility of broad consent under the GDPR (perspective 3)

Even if the EDPB were to push forward and confirm interpretations of consent in the GDPR which would be restrictive or prohibitive to broad consent, an optimistic outlook for broad consent might still be offered based on the practical interplay of data protection law, genomic research and broad consent. Three points deserve further elaboration: first, restrictive data protection law was not necessarily prohibitive to broad consent prior to the GDPR; second, there is no reason to think the enforcement context should change under the GDPR; and finally, even if the two previous points prove incorrect, the genomic research community is well placed to mount resistance.

First, even if the EDPB were to confirm a position which would outright exclude the possibility for broad consent, this would merely constitute a return to the EU data protection legal position prior to the GDPR – positions which would place limits and supplemental requirements on, but would essentially permit, broad consent, would represent an improvement on the situation prior to the GDPR. Yet, practically, data protection laws prior to the GDPR did not necessarily obstruct the use of broad consent in genomic research. In the text of Directive 95/46 – the forerunner to the GDPR – the scope of consent in relation to genomic research was restricted to specific consent. Articles 8 (2)(a) and 2(h) of the Directive were clear on the matter (European Parliament and Council 1995, Articles 8 (2)(a) and 2(h)). In turn, the Article 29 Working Party were also specific in their guidance on the matter. The Working Party explicitly stated, in relation to the specificity requirement, that a scope as broad as ‘future research’ would not be permissible (Article 29 Working Party 2013, p. 16). Certain Member States passed laws legitimately derogating from Directive 95/46 in relation to scientific research which clarified, in the relevant jurisdiction, the legitimacy of broad consent. Other Member States, however, did not pass such laws. In a number of Member States in which no such derogating laws existed, broad consent was nevertheless in unobstructed use. In the UK, for example, there was no clear national legislative basis making broad consent legitimate in relation to the processing of research subjects’ personal data. Yet, broad consent was, and is, in use. See, for example, the use of broad consent in the high-profile genomic research infrastructure project UK Biobank (UK Biobank 2006, p. 1).

Second, it is perhaps the case that the practise of broad consent was unobstructed under Directive 95/46 due to a lack of effort to enforce illegitimacy. If this was the case, however, there seems little reason to think the situation would change under the GDPR. The key enforcement bodies under the GDPR are Data Protection Authorities (DPAs).^14^ These are the same bodies which were responsible for the enforcement of data protection rules under Directive 95/46. The author knows of no case in which a DPA has moved to prohibit or alter tacitly accepted broad consent practises. Indeed, as Gibbons notes – at least in the UK context – DPAs may choose to stay away from interference in genomic research in general (Gibbons 2012, p. 76). There seem several good reasons for DPAs’ reluctance to engage with genomic research. None of these reasons are substantially undermined by virtue of the fact DPAs now enforce the GDPR as opposed to Directive 95/46 – or implementing national legislation. Four reasons seem particularly plausible. First: DPA activity is often triggered by data subject complaints. Data processing in genomic research has hitherto been a calm and uncontroversial activity. Second: genomic research is an esoteric form of processing. DPAs are unlikely to have staff capable of dealing with its specifics. Third: the legitimacy of genomic research is subject to scrutiny by other, well established and discipline proximate, supervisory bodies – in particular research ethics committees. DPAs may feel no need to encroach on their territory. Finally: DPAs operate in a highly politicised environment. DPAs may have no wish to interfere in the practise of genomic research which has clear normative legitimacy and, as observed by Simon et al., public support (Simon et al. 2013, p. 821).

Finally, even if the EDPB were to insist on an interpretation of consent which would prohibit or seriously impede the use of broad consent, and the above observations as to a laissez-faire disjunct between law and practise prove false, there is a final factor speaking for a bright future for broad consent under the GDPR: research community resistance. There is doubtless strong support for the use of broad consent amongst the genomic research community. This support comes from genomic researchers, as well as from lawyers and ethicists considering the practise (Sheehan 2011, p. 226). In turn – see section 1 – this support is, practically, increasingly mirrored in other law on genomic research and in the construction and operation of genomic research infrastructures – population biobanks, for example. Indeed, one might now legitimately argue that broad consent is built into the DNA of European genomic research endeavours (Steinsbekk et al. 2013, p. 897). Any restrictions on the legitimacy or utility of broad consent are thus likely to cause significant cognitive and practical disruption in relation to both existing genomic research infrastructures and future genomic research endeavours. It seems highly unlikely that genomic research organisations – and others with interests tied up with the success of these organisations – would simply accept such an awkward outcome. In this regard, all elements are in place for a forceful resistance: the genomic research community did not simply accept problematic limitations on broad consent in the legislative process leading up to the GDPR (Thompson 2016); the political power of the genomic research community has been proven – as discussed in section 3, their lobbying activity was influential in the legislative process leading up to the GDPR; strong normative and legal arguments are available to the genomic research community – see sections 4 and 5; and, finally, clear judicial avenues already exist through which to mount resistance efforts – Article 78 of the GDPR, for example, offers recourse options against DPAs’ judgments.

The previous three sections have outlined reasons for ongoing optimism as to the legitimacy and utility of broad consent under the GDPR. Despite seemingly negative guidance from the Article 29 Working Party, the future for broad consent in genomic research looks bright.

## Conclusion

In 2017, the Article 29 Working Party released their guidelines on consent in the GDPR. Since then, there has been concern in the genomic research community about the continued legitimacy and utility of broad consent. This concern is understandable. In the first instance, the GDPR is a key instrument of genomic research regulation in Europe and its position on broad consent is thus highly significant. In turn, the content of the Article 29 Working Party guidelines is not friendly to broad consent and the Article 29 Working Party – now European Data Protection Board – are a key entity tasked with providing authoritative interpretations of the GDPR. Whilst recognising the legitimacy of these concerns, this article adopted a more positive outlook and explained why broad consent in genomic research still has a bright future under the GDPR. This outlook was justified from three perspectives.

First, there is a clear overlap between the base rationale behind consent in the GDPR and the base rationale behind broad consent in genomic research. Consent in the GDPR seeks to provide individuals with control over their personal data. Broad consent allows research subjects to control whether they want their biological samples and associated data to be used in future unspecified genomic research. It is true that there are legitimate reasons for the legislator to restrict the degree of control offered to individuals over their personal data under the GDPR. These legitimate reasons are, however, not obviously relevant in relation to genomic research infrastructures operating broad consent. At the same time, the increasing empirical evidence of the social benefits brought by genomic research mitigates against limiting the scope of consent to the detriment of research outcomes.

Second, despite Article 29 Working Party guidance, the legal situation can still be read as conducive to the legitimacy of broad consent. In the first instance, a detailed consideration of the Article 29 guidelines reveals a sweeping vagueness. This vagueness provides the space for legitimate interpretations of the guidance to be put forward which are unproblematic for broad consent. In turn, genomic research is a unique form of scientific research and the GDPR’s rules are designed to be adapted to each case as necessary. Accordingly, the Article 29 Working Party’s general guidance on consent in scientific research might legitimately be set aside if this proves unsuitable and obstructive in relation to the unique practises of genomic research. Finally, even if problematic aspects of the guidance cannot be interpreted away, it may still be argued that the powers of the Article 29 Working Party and the EDPB do not extend to offering interpretations of the GDPR which contradict the express wishes of the legislator. The text of the GDPR suggests the legislator aimed to support broad consent.

Finally, the practice of broad consent has not always been commensurate with the law on broad consent. Even if the EDPB were to confirm interpretations of consent in the GDPR which would be restrictive or prohibitive to broad consent, this would only signal a return to the situation concerning broad consent in EU data protection law prior to the GDPR – under Directive 95/46. Yet, broad consent flourished in many EU Member States despite Directive 95/46 – including in Member States in which no derogating law legitimating broad consent existed. This flourishing may have simply been the result of a lack of enforcement on the part of DPAs. Even if this was the case, however, it is hard to see why the GDPR should lead to a change of course in DPA enforcement strategy. Finally, even if the EDPB were to push forward with a restrictive approach to broad consent and DPAs began to enforce the approach, the genomic research community would still have options, and have already shown their capacity, in mounting resistance.

## Data Availability

Not applicable.

## Footnotes

1 The author declares no competing interest in this article.

2 Whilst this is an article written from a legal perspective, every effort has been made to ensure that the argumentation, and the points made, are generally accessible.

3 Even the most modest interpretations of the scope of the GDPR’s applicability to genomic research only serve to exclude applicability to biological samples.

4 The range of data qualifying as sensitive are exhaustively outlined in Article 9 (1). These include data concerning health and genetic data. The extensive interpretation of the concept of data concerning health means all samples and data used in genomic research will fall within the scope of the concept (Article 29 Working Party 2015, pp. 2–5).

5 Under the GDPR, Article 6 (1) outlines a set of general justifications for the processing of personal data. There is an ongoing debate in data protection law as to the relationship between the justifications in Article 6 (1) and those in Article 9 (2). Specifically: does the legitimation of processing of sensitive personal data require both a legitimation under Article 6 (1) as well as a legitimation under Article 9 (2), or is a justification under 9 (2) alone enough? In other words: is Article 9 (2) a supplemental specification of 6 (1), or is it a stand-alone Article outlining specific and sufficient conditions related to the justification of the processing of sensitive personal data? This article is not the place to mount an extensive effort at substantially engaging in this debate. Nevertheless, a brief discussion as to how the issue relates to the topics dealt with is justified. In this regard, the author would make the initial observation that the issue only becomes a point of legal and practical significance where a justification for processing can be found under Article 9 (2) but not under Article 6 (1) – otherwise there will be no legally relevant lack of correspondence between Articles and no issue. Significantly, this problem constellation is not identifiable in relation to the processing of sensitive personal data in genomic research. This assertion has certain jurisprudential support – at least in relation to Articles 9 (2)(a) and (j). In their recent opinion on the processing of sensitive personal data in clinical trials, for example, the European Data Protection Board suggested the processing of sensitive personal data in research under Articles 9 (2)(a) and (j) both find corresponding encompassing justifications under Article 6 (1) (European Data Protection Board 2019, p. 9). Accordingly, this article – whilst not discounting the argument that a justification under Article 6 (1) may also, in principle, be required – will restrict its discussion of justifications for the processing of sensitive personal data in genomic research to those outlined under Article 9 (2).

6 There is a general caveat to the applicability of Article 9 (2)(a) worthy of note: that there is EU or Member State law applicable which elaborates that the data subject cannot consent to the processing activity in question. As far as the author is aware, however, there are currently no such Union or Member State laws generally relevant to genomic research.

7 There is an interpretation of the GDPR which suggests that Article 9 (2)(a) cannot be used to legitimate publicly supported research. See, for example: https://www.hra.nhs.uk/planning-and-improving-research/policies-standards-legislation/data-protection-and-information-governance/gdpr-guidance/what-law-says/consent-research/. This is not an interpretation which finds extensive support in either jurisprudence or scholarship as regards genomic research. This article is not the place to mount an extensive rebuttal of the applicability of the interpretation as regards genomic research. Nevertheless, a brief discussion – not least to show why the interpretation is not further engaged with – is justified. The interpretation appears to be based on two arguments. First: that the GDPR prohibits consent when ‘[there is an] imbalance of power in the relationship between the controller and the data subject, eg where the controller is a public authority and the data subject depends on their services, or fears adverse consequences, so feels they have no choice but to agree’. This argument largely recites Recital 43 of the GDPR. Recital 43 also states, however, that any consideration of power imbalance and dependence must be taken in relation to ‘a specific case’. The Recital thus does not outline a general prohibition on the use of consent under the GDPR by public bodies, or publicly supported entities – or indeed any specific type of entity. In terms of specifics, it is hard to think of any modern European genomic research infrastructure, or project, which would fulfil the criteria of Recital 43 – these are characterized, as far as they do rely on consent, by their completely optional nature and the fact that participation is unrelated to reciprocity in the provision of current, or future, goods or health-care. Second: that certain data subject rights associated with consent under the GDPR cannot be applied in research and therefore that consent itself under the GDPR should not be used as a legitimation of research. The precise rights in question are not elaborated. It would seem a reasonable assumption, however, that the argument – at least to have any purchase in relation to genomic research – refers particularly to the right to withdraw consent, under Article 7 (3), and the consequent right to the erasure of personal data following withdrawal of consent, under Article 17 (1)(b). The right to have personal data erased following a withdrawal of consent would indeed be problematic for genomic research if the right were absolute. In this case, how could genomic data sets’ integrity be ensured and how could research be effectively validated? The right to erasure as it relates to genomic research, however, is not absolute. In this regard, the right must be read in conjunction with the applicable exception outlined in 17 (3)(d) – also applicable when consent under 9 (2)(a) is the ground used to legitimate processing – which permits retention of personal data for research despite withdrawal of consent if erasure: ‘is likely to render impossible or seriously impair the achievement of the objectives of [the research].’

8 The concept of a ‘substantial’ public interest has no independent meaning in EU law and no definition is provided in the text of the GDPR. There is jurisprudence available, however, in relation to the seemingly comparable concept of an ‘important’ public interest. In this regard, the Article 29 Working Party observed that: ‘[the concept of an] ‘important public interest [should be] given a restrictive interpretation’ and should refer to processing which is ‘necessary and…identified as [an important public interest] by…national legislation’ (Article 29 Working Party 2005, pp. 14–15).

9 Elsewhere in the GDPR, further relevant criteria for such laws are outlined. Recital 41, in particular, clarifies criteria concerning the forms – the types and levels of law – and qualities – the procedural and substantive standards to which law must adhere – of laws capable of triggering Articles 9 (2)(g) and (j).

10 There is one obvious exception to the validity of these legal arguments asserting the legal primacy of Article 9 (2)(a): if Member State legislation under 9 (2)(g) or (j) also highlights consent as the primary means to legitimate genomic research. In this case, there is no reason why the consent outlined in Article 9 (2)(a) should be regarded as legally superior to that outlined in Member State law subsequent to 9 (2)(g) or (j).

11 More work remains to be done on how different Member State legal systems have categorized the concept of a ‘substantial’ public interest as opposed to an ‘ordinary’ public interest as well as on how different Member States treat the possibility to rely on Article 9 (2)(g) as a justification for genomic research.

12 For example, what precisely is meant by the phrase ‘recognised ethical standards’ – local, national, international?

13 Supplemental to these proclamations, the Working Party then clarify, in cases Recital 33 is relied upon, that a series of supplementary safeguards should be in place to make up for any deficit caused by a lack of clarification of a specific project in the initial consent transaction. The Working Party suggest that these safeguards might include: transparency measures to ensure research subjects always have relevant information concerning the scope of the project; substantive measures concerning the minimisation of the identifiability of samples and personal data used in the project; and substantive measures ensuring adherence to data protection by design and default provisions in the information processing systems used in the project (Article 29 Working Party, 2017, p. 28).

14 National supervisory authorities mandated to ensure the provisions of the GDPR are followed – see Chapter VI GDPR.
